# Emerging evidence for anti-PD-1 and IFN-γ as adjunctive immunotherapy in invasive mold infections

**DOI:** 10.1128/mbio.00230-26

**Published:** 2026-05-22

**Authors:** Alexandra Serris, Amélie Guihot, Jeremie Joffre, Julien Dessajan, Laureen Dahuron, Alexie Bosch, Emmanuel Dudoignon, Lucien Pierot, Lucie Lelièvre, Anne Claire Lukaszewicz, Guillaume Monneret, Olivier Lambotte, Guillaume Martin-blondel, Fanny Lanternier

**Affiliations:** 1 Infectious Diseases Department, Universitary Hospial Necker-Enfants Malades, Assistance Publique-Hôpitaux de Paris, Paris Cité University 555089, Paris, France; 2 Translational Immunology Unit, Institut Pasteur, Université Paris Cité 555089, Paris, France; 3 Sorbonne Université, Inserm, U1135, CNRS ERL8255, Centre d'Immunologie et des Maladies Infectieuses (CIMI-Paris) 27051, Paris, France; 4 Immunology Department, Universitary Hospital Pitié-Salpêtrière, Assistance Publique-Hôpitaux de Paris 26930, Paris, France; 5 Medical Intensive Care Unit, Hôpital Saint-Antoine, Assistance Publique-Hôpitaux de Paris, Sorbonne Université 27063, Paris, France; 6 Inserm UMRS 938—Immune System and Neuro-Inflammation, Hôpital Saint-Antoine 37117, Paris, France; 7 Medical and Infectious Diseases ICU, Paris Cité University—Bichat University Hospital, Assistance Publique—Hôpitaux de Paris 555089, Paris, France; 8 Infectious Diseases Department, Regional Universitary Tours Hospital, Tours, France; 9 Infectious Diseases Department, Chambéry Hospital, Chambéry, France; 10 Department of Anesthesiology and Critical Care and Burn Unit, Groupe Hospitalier St Louis-Lariboisière, Assistance Publique—Hôpitaux de Paris (AP—HP), Université Paris-Cité 555089, Paris, France; 11 Institut National de la Santé et de la Recherche Médicale (INSERM), INSERM UMR-S 942 Mascot, Lariboisière Hospital, Paris, France; 12 FHU PROMICE, Paris, France; 13 Department of Anesthesiology and Critical Care, Rangueil University Hospital, Toulouse, France; 14 Department of Infectious and Tropical Diseases, Toulouse University Hospital, Toulouse, France; 15 Department of Anesthesiology and Critical Care, Neuroscience Research Center, Hospices Civils de Lyon, Hôpital Neurologique Pierre Wertheimer and Université Lyon 1 26900, Lyon, France; 16 Immunology Laboratory, Hospices Civils de Lyon 26900, Lyon, France; 17 Department of Internal Medicine and Clinical Immunology, AP-HP, GHU Paris Saclay, Le Kremlin Bicêtre, France; 18 Université Paris-Saclay, Inserm, CEA, Center for Immunology of Viral, Auto-immune, Hematological, Bacterial Diseases (IMVA-HB/IDMIT/UMRS1184), Le Kremlin Bicêtre, France; 19 IHU Sepsis Comprehensive Center, Garches, France; 20 Toulouse Institute for Infectious and Inflammatory Diseases (Infinity), INSERM UMR1291, CNRS UMR5051, Toulouse III University, Toulouse, France; 21 Neo-I3D Research Group, Toulouse University Hospital, Toulouse, France; 22 National Reference Center for Invasive Mycoses and Antifungals, Translational Mycology Research Group, Mycology Department, Institut Pasteur, Université Paris Cité 555089, Paris, France; Medizinische Universitat Graz, Graz, Austria

**Keywords:** invasive fungal diseases, mucormcyosis, immunotherapy, anti-PD-1 mAbs, IFN-γ

## Abstract

Invasive mold diseases (IMDs) such as mucormycosis and aspergillosis carry high mortality despite optimal antifungal therapy. Adjunctive immunomodulation, including anti-PD-1 antibodies and interferon-γ, may help restore antifungal immunity in severely ill patients. We implemented multidisciplinary team meetings in France to assess and validate the use of adjunctive anti-PD-1 monoclonal antibodies and interferon-γ in combination with antifungal therapy for patients with life-threatening IMDs. Here, we report the characteristics and outcomes of the treated patients. Twelve cases were reviewed, and eight patients ultimately received adjunctive therapy. All presented with particularly severe IMDs, including seven mucormycosis (three cerebral, three intra-abdominal, and one extensive fasciitis) and one pulmonary aspergillosis. PD-1 expression on T cells was elevated in all patients. Following adjunctive treatment, six of the eight patients survived. Therapy was generally well tolerated; serious adverse events included one episode of cutaneous toxicity and two cases of acute respiratory distress syndrome, possibly related to immunotherapy. All resolved after treatment discontinuation. In our experience, adjunctive immunotherapy combined with antifungals was mostly associated with favorable outcomes in severe, refractory IMDs. These findings support further investigation of host-directed strategies as potential adjuncts in managing life-threatening fungal diseases.

**IMPORTANCE:**

This study provides preliminary evidence supporting the clinical relevance of host-directed immunotherapy as an adjunct to antifungal treatment in severe invasive mold diseases (IMDs), which remain associated with high mortality despite optimized antifungal regimens. By targeting immune dysfunction, particularly T-cell exhaustion mediated through the PD-1 pathway, the combined use of anti-PD-1 monoclonal antibodies and interferon-γ aims to restore effective antifungal immune responses. The observed survival benefit in this small cohort supports the biological rationale that reversing immune paralysis can enhance pathogen clearance in patients with IMDs. Although limited by sample size, this work provides encouraging evidence supporting the feasibility, tolerability, and potential efficacy of combined immunomodulatory strategies, thereby contributing to the evolving paradigm of personalized and immune-guided management in IMDs. Future work should focus on biomarker-guided patient selection, rigorous monitoring, and determining the optimal timing for therapy initiation. Integrating immunological profiling into patient assessment may enable more precise, stratified therapeutic approaches.

## INTRODUCTION

Invasive fungal diseases (IFDs) remain associated with high morbidity and mortality, despite significant advances in diagnostics and the availability of effective antifungal agents. Emerging evidence implicates T-cell exhaustion and broader immune dysregulation as key contributors to IFDs pathogenesis, both in immunocompromised and immunocompetent individuals (1, 2). In this context, adjunctive immunotherapeutic strategies aimed at restoring immune competence have gained increasing attention.

A variety of immunomodulatory interventions—cellular and non-cellular—have been explored in animal models to enhance antifungal immunity. In humans, immunomodulatory therapeutic options remain limited. Among these, recombinant interferon gamma (rIFN-γ) has shown the ability to restore immune function in patients with IFDs (3), enhancing macrophage activation and increasing major histocompatibility complex (MHC) gene expression on antigen-presenting and target cells. However, its limited tolerability has restricted widespread use. More recently, immune checkpoint blockade (ICB) has emerged as a promising strategy, with several monoclonal antibodies available for therapeutic targeting. In particular, inhibition of the programmed cell death protein 1 (PD-1) pathway has become the most widely used form of ICB. Engagement of PD-1 with its ligand PD-L1/PD-L2 initiates a negative co-inhibitory signal that results in T-cell dysfunction, exhaustion, and immune tolerance. ICB monoclonal antibodies targeting PD-1 or PD-L1 can disrupt this interaction, thereby reversing T-cell exhaustion and reactivating T-cell functions, reinvigorating the immune response to the pathogen. Anti-PD-1 and PD-L1 are now broadly used in oncology against a wide range of tumors, and their safety profiles are well established. Their use in IFDs could lead to the restoration of antifungal effector functions. Indeed, preclinical studies in murine models of aspergillosis (4), mucormycosis (5), cryptococcosis (6), and paracoccidioidomycosis (7) have demonstrated that PD-1 blockade restores T-cell functions, augments cytokine production (e.g., IFN-γ and IL-17A), enhances fungal clearance, and improves survival outcomes. To date, seven clinical case reports have documented the use of anti-PD-1 monoclonal antibodies, most often combined with rIFN-γ in the management of IFDs (8–14).

We initiated dedicated multidisciplinary team meetings (MDTs) affiliated with the National Reference Center for Invasive Fungal Diseases in France to discuss the indication of immunotherapy in addition to the standard of care in severe invasive mold infections. We report the experience of the patients evaluated in these MDTs, with one additional case that was evaluated in another MDT and treated in the intensive care unit of HCL Hospital (11).

## MATERIALS AND METHODS

**Fig 1:**
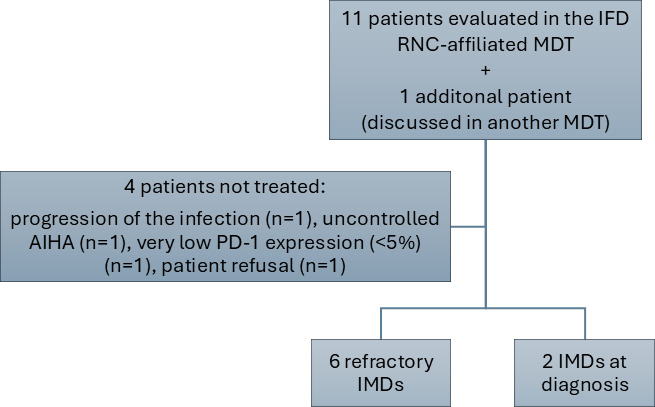
Flowchart of the patients evaluated for immunotherapy of IFDs in MDTs. IFD, invasive fungal diseases; RNC, reference national center; MDT, multidisciplinary team meetings. AHAI, autoimmune hemolytic anemia; IMDs, invasive mold diseases.

Twelve potentially eligible patients were discussed over a 2-year period. The decision considered the severity and location of the infection (e.g., brain or abdominal infection), the response to antifungal therapy, the feasibility of surgical infection control, the presence of comorbidities, and, when rapidly available, PD-1 expression on CD4 and CD8 T cells. Of the 12 patients discussed, eight received immunotherapy (Fig. 1). The remaining four were not treated because of rapid death due to the progression of the infection (*n* = 1), uncontrolled autoimmune anemia (*n* = 1), very low PD-1 expression (<5%) on circulating T cells (*n* = 1), and patient refusal (*n* = 1).

We monitored the level of PD-1 expression at T lymphocyte surface and evaluated the therapeutic antibody fixation on T cells, according to a pre-established flow cytometry protocol (15).

## RESULTS

The clinical characteristics of the eight patients are summarized in Table 1. Patients #1, #2, and #4 have previously been described in individual case report publications (10, 11, 14).

**TABLE 1:** Patients’ characteristics: invasive mold diseases, underlying conditions, and antifungal treatment^*a*^

Id (reference)	Age and gender	Invasive mold disease risk factors	Invasive mold disease	Antifungal treatment	Surgical resection of infected tissues	Outcome
#1 (10)	M, 56 yrs	– Uncontrolled type 2 diabetes mellitus – Severe SARS-CoV-2 infection treated with corticosteroids and anti-IL6 mAbs	– COVID-associated pulmonary aspergillosis (CAPA) due to *Aspergillus flavus* – Rhino orbital cerebral mucormycosis due to *Rhizopus* sp. with positive culture of brain abscess and positive Mucorales PCR in serum	L-Amb associated with isavuconazole	Yes (drainage of one of the cerebral abscesses)	Death (day 68)
#2 (11)	F, 38 yrs	Septic shock secondary to extensive streptococcal fasciitis	Mixed fasciitis due to *Aspergillus fumigatus*, *Aspergillus terreus*, *Lichtheimia ramosa*, and *Rhizopus arrhizus* with positive tissue cultures and positive Mucorales PCR in serum	L-Amb associated with an azole (posaconazole then isavuconazole)	Yes	Alive without antifungal treatment—at home (follow-up: 140 days)
#3	M, 69 yrs	Ethmoidal adenocarcinoma treated by surgery and radiotherapy	Rhino-orbital cerebral mucormycosis with meningitis due to *Rhizopus* sp., with positive Mucorales PCR in the CSF and positive culture of sinus samples	L-Amb associated with an azole (posaconazole then isavuconazole) and caspofungin	No	Alive under isavuconazole alone—at home (follow-up: 614 days)
#4 (14)	M, 68 yrs	– Type 2 diabetes mellitus (well equilibrated) – Invasive sigmoid adenocarcinoma treated with neoadjuvant chemotherapy and surgery – Multiple abdominal surgeries for nosocomial bacterial peritonitis – Nosocomial bacterial peritonitis	Cutaneous, peritoneal, and intestinal mucormycosis due to *Rhizopus microsporus* with positive histological examination of digestive biopsies and Mucorales PCR in serum	L-Amb associated with isavuconazole and caspofungin	Yes	Alive without antifungal treatment—at home (follow-up: 405 days)
#5	M, 63 yrs	– Multiple myeloma treated with CAR-T cell injection 2 months before IFD diagnosis complicated with grade 2 CRS and ICANS treated with steroids, anti-IL1, anti-IL6 – Severe flu infection	Influenza-associated pulmonary aspergillosis due to susceptible *A. fumigatus* with positive galactomannan and *Aspergillus* PCR in serum	Voriconazole associated with caspofungin	No	Death (day 13)
#6	F, 36 yrs	– Septic shock secondary to group A *Streptococcus* peritonitis following emergency cesarean section – Multiple abdominal surgeries	Cutaneous and peritoneal mucormycosis due to *Lichtheimia* sp. with positive culture of digestive biopsies	L-Amb associated with isavuconazole (later switched to posaconazole alone)	Yes	Alive under posaconazole treatment—in a rehabilitation center (follow-up: 238 days)
#7	M, 25 yrs	Multiple traumatic injuries and extensive electrical burns	Cutaneous, peritoneal, and bone mucormycosis due to *Lichtheimia ramosa*	L-Amb associated with isavuconazole	Yes	Alive without antifungal treatment—in a rehabilitation center (follow-up: 201 days)
#8	M, 39 yrs	Uncontrolled type 2 diabetes mellitus	Rhino-orbito-cerebral mucormycosis due to *Rhizopus arrhizus*	L-Amb associated with isavuconazole	Yes	Alive under isavuconazole treatment—in a rehabilitation center (follow-up: 93 days)

^*a*^ mAbs, monoclonal antibodies; L-Amb, liposomal amphotericin B; rIFN-γ, recombinant interferon gamma; CSF, cerebrospinal fluid; ICANS, immune cell-associated neurotoxicity syndrome; IFD, invasive fungal disease.

Among the eight patients, three were previously immunocompetent but likely developed an immune-paralysis state associated with septic shock (16), two had poorly controlled diabetes mellitus, two had underlying neoplasms, and one had multiple myeloma treated with CAR-T cells.

Each patient demonstrated extensive tissue invasion and posed major therapeutic challenges. Patient #1 presented with COVID-associated pulmonary aspergillosis due to *Aspergillus flavus* together with rhino-orbito-cerebral mucormycosis (ROCM) caused by *Rhizopus* spp. that did not respond to a dual therapy with liposomal amphotericin B (L-AmB) and isavuconazole, as well as drainage of one of the brain abscesses (the second being inaccessible to surgery). Patient #2 had mixed fungal fasciitis of the left chest wall, due to *Aspergillus fumigatus*, *Aspergillus terreus*, *Lichtheimia ramosa*, and *Rhizopus arrhizus*, and despite treatment with L-AmB and posaconazole, and repeated surgeries, infection spread to the surface and the pleura, leading to a pneumothorax with tissue biopsies remaining persistently culture positive. Patient #3 developed rhino-orbito-cerebral mucormycosis with meningitis due to *Rhizopus* spp. and showed continued progression despite combination therapy with L-AmB, isavuconazole, and caspofungin, with worsening meningeal extension. Patient #4 exhibited cutaneous, peritoneal, and intestinal mucormycosis caused by *Rhizopus microsporus* and deteriorated despite combined therapy with L-AmB, isavuconazole, caspofungin, along with repeated surgical debridement of necrotic lesions of the abdominal wall and peritoneum. Patient #5 had influenza-associated pulmonary aspergillosis due to *A. fumigatus* and failed to improve under dual therapy with voriconazole and caspofungin. Patient #6 suffered from cutaneous and peritoneal mucormycosis due to *Lichtheimia* spp. and received L-AmB, isavuconazole, associated with extensive surgical debridement, though the sheer extent of the lesions prevented adequate surgical control of the infection. Patient #7 presented with cutaneous, peritoneal, and bone mucormycosis caused by *Lichtheimia ramosa* and was treated with L-AmB, isavuconazole, and surgery; however, the widespread involvement similarly precluded effective surgical source control. Patient #8 had rhino-orbito-cerebral mucormycosis caused by *Rhizopus arrhizus*, progressing despite L-AmB, isavuconazole, and extensive surgical resection, with new brain lesions developing during therapy.

Therefore, immunotherapy was introduced after failure of a well-conducted antifungal treatment combined with surgical excision of infected tissue when feasible in six patients and at the time of invasive mold disease (IMD) diagnosis due to extreme severity in two patients (#6 and #7: both had extensive intra-abdominal mucormycosis lesions not amenable to surgical resection; Table 1).

All patients received initial immunotherapy combining anti-PD-1 mAbs and rIFN-γ, except for the patient recently treated with CAR-T cells, in whom rIFN-γ was deemed too unpredictable. In patients with refractory IMD, the median duration of antifungal treatment before anti-PD-1 mAbs infusion was 16 days (15–20). At immunotherapy onset, median neutrophil and lymphocyte counts were 8.7 G/L (5.2–13.2) and 0.8 (0.6–2), respectively. None of the patients had a neutrophil count below 1 G/L.

**Fig 2:**
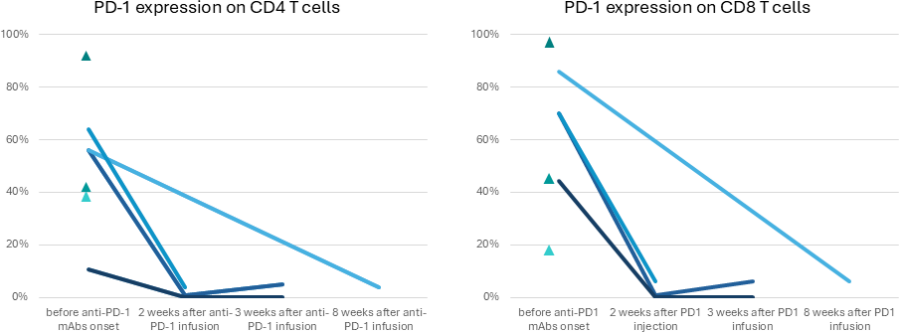
Expression levels of PD-1 on circulating CD4^+^ and CD8 T cells. Each color represents the same patient.

The type of immunotherapy is detailed in Table 2. PD-1 expression on circulating CD4^+^ and/or CD8^+^ T cells was elevated before treatment in most patients, with a median of 56% (40–60) for CD4^+^ T cells and 70% (40–80) for CD8^+^ T cells (Fig. 2). Most patients received a single anti-PD-1 infusion. PD-1 expression remained low 2 weeks post-infusion in four patients (<5%) and persisted at low levels for up to 8 weeks in one patient (Fig. 2).

**TABLE 2:** Characteristics of the immunotherapy protocols^*b*^

Id	Delay between anti-PD-1 injection and antifungal treatment initiation (days)	Immunotherapy	Number of anti-PD-1 perfusions	Duration of rIFN-γ therapy (days)
#1	14	Nivolumab 240 mg plus rIFN-γ 100 µg × 3/week	2	30 days
#2	17	Nivolumab 280 mg plus rIFN-γ 100 µg × 3/week^*a*^	1	10 days
#3	92	Nivolumab 240 mg plus rIFN-γ 100 µg × 3/week	4	42 days
#4	21	Nivolumab 240 mg plus rIFN-γ 100 µg × 3/week^*a*^	1	10 days
#5	15	Nivolumab 240 mg	1	-
#6	5	Nivolumab 240 mg plus rIFN-γ 100 µg × 3/week	1	19 days
#7	2	Nivolumab 240 mg plus rIFN-γ 100 µg × 3/week	1	30 days
#8	15	Nivolumab 240 mg plus rIFN-γ 100 µg × 3/week	1	5 days

^*a*^ Patients #2 and #4 had received rIFN-γ for 16 and 7 days, respectively, before anti-PD-1 injection. All other patients received the first injection of anti-PD-1 mAbs and rIFN-γ concomitantly.

^*b*^ Patient #1 received two anti-PD-1 injections 4 weeks apart. Patient #3 received four injections: day 1, day 110, day 167, and day 201.

Despite the severity and refractoriness of these mold infections, six patients of eight survived after a median follow-up of 7.2 months (5.1–11.9). By the end of follow-up, three patients had achieved a cure, with no relapse after discontinuing antifungal therapy, while the remaining three maintained infection control under ongoing antifungal treatment (Table 1). All were discharged from the hospital, with two requiring rehabilitation care for surgical sequelae such as limb amputations and extensive digestive tract resections. Both deaths occurred in severely immunocompromised patients requiring ICU hospitalization: one had recently received CAR-T cell therapy for myeloma, and the other had been treated with high doses of corticosteroids and anti-IL-6 monoclonal antibodies for a severe SARS-CoV-2 infection. In both cases, death was attributed to the progression of the fungal infection, occurring in the context of multiple organ failure, compounded by co-infections and drug toxicities.

Treatment was generally well tolerated. All reported adverse events (AEs) are summarized in Table 3. Their imputability was considered possible in four situations and high in two. One patient developed Drug Reaction with Eosinophilia and Systemic Symptoms (DRESS), 11 days after the anti-PD-1 infusion, in the context of multiple concomitant medications. Although the exact causative agent could not be determined, anti-PD-1 therapy was not re-administered. The DRESS syndrome resolved rapidly following the discontinuation of all suspected drugs and the initiation of topical corticosteroid treatment. Another patient—who was being treated for refractory pulmonary aspergillosis associated with influenza following recent CAR-T cell therapy—developed an acute respiratory distress syndrome (ARDS) 10 h after receiving the anti-PD-1 injection. Although severe immune-related adverse events (irAEs) reported in the literature typically occur later after immune checkpoint inhibitors (ICI) initiation, with a median onset of 36 days (14–98) for respiratory system AEs (17), an immune reconstitution syndrome triggered by anti-PD-1 mAbs administration cannot be ruled out. This same patient also presented a rapidly resolving myocarditis with transient left ventricular dysfunction and troponin elevation, with concomitant parvovirus B19 viremia. rIFN-γ was less well tolerated and was associated with fever and febrile neutropenia in three patients, leading to its discontinuation. One of the febrile patients—who was hospitalized in ICU for a refractory ROCM—also developed an ARDS 3 days after the onset of immunotherapy. No bacterial infection was documented, and the patient improved after empirical antibiotic therapy and suspension of rIFN-γ therapy. None of the patients who presented an ARDS were treated with corticosteroids.

**TABLE 3:** Reported adverse events possibly related to immunotherapy^*a*^

Adverse event	Number of events reported (patient’s ID)	Grade	Suspected molecule	Delay between onset of treatment and first symptoms	Other possible factors	Evolution	Imputability
DRESS	1 (#4)	3	Nivolumab	11 days	Multiple concomitant comedications	Resolution after interruption of all imputable treatments	Possible
ARDS	1 (#5)	4	Nivolumab	10 h	Intercurrent bacterial infection	Improvement after empirical antibiotherapy	Possible
Myocarditis	1 (#5)	3	Nivolumab	5 days	Parvo B19 infection	Rapid resolution (the Parvo B19 infection was treated with IgIV)	Possible
Febrile neutropenia	1 (#7)	4	rIFN-γ	30 days	Multiple concomitant comedications	Resolution after interruption of all imputable treatments	High
Fever	2 (#3 and #8)	2–3	rIFN-γ	5 and 42 days, respectively	None	Resolution after rIFN-γ interruption	High
ARDS	1 (#8)	4	rIFN-γ/PD-1	3 days	Intercurrent bacterial infection	Improvement after empirical antibiotherapy and rIFN-γ interruption	Possible

^*a*^ DRESS, drug reaction with eosinophilia and systemic symptoms; ARDS, acute respiratory distress syndrome.

## DISCUSSION

Our study provides encouraging evidence supporting PD-1 as a therapeutic target in IMD, particularly in mucormycosis. In most cases, anti-PD-1 therapy was administered in combination with rIFN-γ, based on the hypothesis of a potential synergistic effect between immune checkpoint inhibition and cytokine-based immunomodulation. The duration of rIFN-γ treatment varied widely, and its discontinuation due to drug-related adverse events did not appear to negatively affect survival. It may be hypothesized that the rapid mechanism of action of rIFN-γ is particularly beneficial at the start of treatment, while awaiting the onset of anti-PD-1 monoclonal antibody effects. Recombinant IFN-γ therapy has been shown to enhance leukocyte immune responses to fungal infections by restoring antigen presentation through increased MHC II expression, promoting secretion of pro-inflammatory cytokines by the innate immune system (such as IL-1β and TNF-α), and increasing the production of T-cell-derived cytokines, including IL-17 and IL-22 (3, 18). In preclinical models of IFDs, ICI therapy has also been associated with multiple immunological effects, including increased recruitment of innate immune cells, restoration of antigen-presenting cell (APC)-T-cell interactions, enhanced maturation and fungicidal activity of APCs, reversal of T-cell exhaustion with improved T-cell activation, and increased cytokine production (19). These broad effects may be explained by the role of the PD-1/PD-L1 pathway in regulating not only T-cell responses but also other leukocyte populations, including natural killer cells and mononuclear phagocytes. Therefore, combining these approaches may be complementary, targeting both the innate and the adaptive immune responses to the pathogen. Moreover, a preliminary study in oncology reported that combining IFN-γ with anti-PD-1 monoclonal antibodies led to greater T-cell proliferation and cytolytic activity *in vitro* compared with anti-PD-1 therapy alone (20). Some authors have hypothesized that the addition of IFN-γ could overcome resistance to PD-1 blockade observed in certain tumors via activation of cytotoxic Foxp3^−^CD4^+^ and CD8^+^ T cells and NK cells (21).

Experience from oncology indicates that anti-PD-1 mAbs can induce irAEs such as pneumonitis, colitis, endocrinopathies, and myocarditis, with severe events being relatively uncommon and late (median onset time 1–3 months, depending on the organ involved) (22, 23). In infectious diseases, additional risks include immune reconstitution inflammatory syndrome, which may precipitate or worsen organ dysfunction at the site of infection. In our series, the imputability of the adverse events to the anti-PD-1 remains difficult to assess due to other possible causes. The onset of irAEs may also differ from that reported in oncology, possibly due to the distinct patterns of persistent inflammation and immune suppression in these conditions. Indeed, preclinical studies have reported signs of ICI-related immunotoxicity in settings of acute infection characterized by hyperinflammation and high antigenic load (4). In contrast, a phase 1b trial evaluating anti-PD-1 therapy in bacterial sepsis reported no safety concerns (22). Some studies in animal models also suggested that reduced dosing (compared with that used in oncology) may substantially mitigate toxicity while still enhancing fungal clearance (4). Use of low doses of anti-PD-1 in humans has been associated with a reduced risk of irAEs and restoration of efficient anti-HIV T cell responses (24). However, the small sample sizes in infectious disease studies of anti-PD-1 therapy in humans currently prevent definitive safety conclusions, and patients receiving immunotherapy for IFDs should be carefully monitored. In our study, rFN-γ was associated with transient fever and flu-like symptoms, in accordance with previous reports in the literature (25). Interestingly, in a small series of kidney transplant recipients, rIFN-γ administration was not associated with an increased risk of allograft rejection (26).

PD-1 expression levels in our study were comparable to those reported in a study analyzing lymphocyte immune phenotypes in 30 patients with either invasive aspergillosis or mucormycosis (1). In another invasive fungal disease, invasive candidiasis, elevated PD-1 expression on T cells has been associated with increased mortality (2). Although PD-1 expression may serve as a marker of poor prognosis, prompting us to propose an immunomodulatory treatment in addition to antifungal therapy, it remains uncertain whether it reliably predicts responses to anti-PD-1 mAbs. In oncology, PD-1 expression on tumor-infiltrating CD8^+^ T cells appears to be a useful predictor of therapeutic response, but the evidence regarding circulating PD-1^+^ T cells remains inconsistent (27). In infectious diseases, tissue biopsies are rarely performed, highlighting the need for future studies to identify blood-based predictors of response to anti-PD-1 therapy. A more refined immunophenotyping approach that distinguishes between different exhausted T cell subsets (e.g., progenitor TCF1^+^ versus terminally differentiated T cells) may provide greater accuracy than the phenotype we assessed (28). A better understanding of the pathophysiology of these severe infections is essential to optimize their management, including decisions regarding the addition of rIFN-γ, as well as the number and dosing of anti-PD-1 infusions.

Most IFD risk factors identified in our cohort, namely diabetes, sepsis, and trauma, are conditions associated with known moderate or transient impairments in antifungal immunity (29). Our findings suggest that, in this context, defects in innate and/or adaptive immune responses may be restored through immunostimulatory therapies, as previously demonstrated for patients with bacterial sepsis (30). In the context of sepsis, immunotherapies targeting distinct immune phenotypes, such as anti-IL-1 for macrophage activation-like syndrome and IFN-γ for sepsis-induced immunoparalysis, have been associated with improvements in organ dysfunction when guided by specific immunological parameters. Indeed, the efficacy of IFN-γ therapy depends on identifying patients who can respond to the cytokine but exhibit impaired endogenous production during infection. In this context, reduced monocyte HLA-DR expression has emerged as a promising biomarker for patient selection (30). Accordingly, a stratified therapeutic approach may also prove beneficial in patients with invasive fungal diseases, using a combination of biomarkers, such as monocyte HLA-DR and PD-1 expression, to guide the selection of immunotherapy (recombinant IFN-γ, anti-PD-1 monotherapy, or combination strategies).

Other major risk factors for IFDs are hematological malignancies with profound neutropenia. Such patients are nearly absent from our cohort, precluding any conclusions for this population. The potential benefit of immunostimulatory therapy in these patients therefore remains to be determined. Anti-PD-1 monoclonal antibody therapy is associated with a high risk of allograft rejection in solid organ transplant recipients, as well as graft-versus-host disease in hematopoietic stem cell recipients, particularly when immunosuppressive therapy is concomitantly reduced—as is often the case in the management of IFDs—as well as with the risk of autoimmune flare, especially in patients with pre-existing autoimmune diseases (31). For these reasons, we did not propose immunotherapy for such patients. We believe that ICI therapy might be considered in this population once the efficacy of immunotherapy is more clearly established for IFDs, as in oncological settings. In such cases, treatment should be evaluated on a case-by-case basis by a multidisciplinary team, with careful patient monitoring and individualized adjustment of immunosuppressive therapy.

Our cohort has several limitations. It is an exploratory case series characterized by a small sample size of patients with heterogeneous clinical conditions, with limited immunological data, making mechanistic and clinical attribution difficult. However, in IMD, immunotherapy with anti-PD-1 monoclonal antibodies and IFN-γ represents a promising adjunct to antifungal therapy, particularly in patients with reversible or moderate immunosuppression, as suggested in other infectious diseases such as JC virus, malaria, HIV infection, and hepatitis B virus infection (32, 33). Future work should focus on biomarker-guided patient selection, rigorous monitoring, and determining the optimal timing for therapy initiation.
